# Deglutition Excited by Dashing Cold Water on the Face

**Published:** 1847-05

**Authors:** 


*Deglutition excited by dashing Cold Water on the Face.*—The following suggestion by Mr. Simpson of Stamford, founded upon the invaluable principles of the excito-motory system is worthy of more general application in cases in which the power of voluntary deglutition is lost. The suggestion is well illustrated in the subjoined case.

A poor man, who had attempted suicide, was sinking from the effects of loss of blood; his pulse was imperceptible, and the action of the heart could scarcely be felt. It being desirable to administer stimulants, his mouth was filled with spirits and water, but the patient was unconscious, and therefore did not swallow. Cold water was dashed upon his face for the purpose of making him swallow by exciting reflex action, when the contents of the mouth were instantly gulped down.—*Lon. Lancet.*—*Ibid.*

